# Physical Diagnosis*Abstract of a Lecture recently delivered at Columbia Veterinary College.

**Published:** 1883-01

**Authors:** E. Benjamin Ramsdell

**Affiliations:** Lecturer on Diseases of the Respiratory Apparatus, Columbia Veterinary College


### ART. III.—PHYSICAL DIAGNOSIS.*

BY E. BENJAMIN RAMSDELL, M.D.,

*Lecturer on Diseases of the Respiratory Apparatus, Columbia Veterinary College.*

TO-DAY we commence the study of diseases of the Respiratory Apparatus. We shall divide our subject into two parts, and during the earlier portion of this course we will devote the hour to Physical Diagnosis; while later on the diseases proper of the lungs and accessory apparatus will be considered.

Physical diagnosis has been defined as "those methods which are employed for detecting disease during life, by the anatomical changes which it has produced."

While the treatment of diseased conditions is the grand object of the physician, still the *diagnosis* of the same must precede the treatment, and therefore is really the more important; for although diagnosis without treatment may have a cure, yet treatment without diagnosis is almost certain to do injury to the suffering animal.

The diagnostician must understand pathology—that is, he must be thoroughly conversant with all the changes which may take place in an organ under the baneful influences of disease. In order to do this he must have a clear conception of the structure and the various functions of the organs under consideration, and therefore must be an anatomist and a physiologist. Remember that in our conflict with disease there are the two important parts of the fight: first, the detection, or diagnosis; and secondly, the correction, or treatment. Observe, I put the detection first, not only on account of the logical, but also on account of its inherent importance.

No one can without fear and timidity, treat cases of whose diagnosis he is ignorant. I do not wish to enter into a scholastic discussion, but I would say that any medical school or

*Abstract of a Lecture recently delivered at Columbia Veterinary College.

system which inculcates the *treatment of symptoms* and ignores the knowledge of the disease itself, is founded not upon a rock, but on sand, and will not be able to withstand the waves of time which will roll about and wash away its insecure foundation.

Diagnosis, then, is the scientific consideration of symptoms. Watson has defined a symptom as " everything or circumstance happening in the body of a sick individual and capable of being perceived by himself or others, which can be made to assist our judgment concerning the seat, or nature of the disease, its probable course and termination, or its proper treatment.

A more laconic answer would be that *symptoms* are the *signs of the disease.* They are important not per se, but only when scientifically considered by a skillful physician.

Just to illustrate the difference between the trained and the untrained observer, I have seen a patient, whose eye had previously been declared unsound, taken before a class of first-course students, men of possibly more than ordinary intelligence. After inspection they were unable to find any trouble in it, and although it was *artificial* none discovered it.

There are two classes of symptoms: first, those which are appreciated and noticed by the patient only; e. g. pain, dyspnœa, etc.; secondly, those which are apprehended by the physician; e. g. rapid pulse, increased temperature, rapid respiration, cough, etc., etc.

The first class has been named the subjective, whereas those observed by the physician have been called the objective symptoms.

In the examination of the dumb animals none but the objective symptoms can be considered. Our diagnosis therefore is dependent upon the careful consideration of these objective symptoms.

Under the general term, physical diagnosis, we have seven methods which we employ in our search: First, Inspection; second, Palpation; third, Percussion; fourth, Auscultation; fifth, Auscultatory Percussion, sixth, Mensuration, and seventh, Succussion. These are named in the order in which they should be employed in the diseases of the Respiratory Apparatus; and to them I would add, as an important aid, examination of the urine, physically, chemically and microscopically.

The first named method, Inspection, is simply the ocular examination of our subject.

By it we appreciate the attitude, the general condition, the number and rhythm of the respiratory acts, etc.

The respiratory act, as you well know, is divided into an inspiration followed immediately by an expiration; and then occurs a period of rest. We first count the number of these respiratory acts per minute. In the horse there are normally twelve to fifteen such respiratory movements per minute.

Many causes may produce an increase in their frequency, and the mere increase in the number of these acts is of little value. It is like a symbol from the alphabet, which by itself is worthless; but when associated and conjoined properly with others of its kind, it goes to make up a word, which is the expression of an idea; the grand object of the writing. So with one symptom, by itself of little value, but grouped with other symptoms, it forms the chain of thought which expresses the diagnosis, the grand object of our search.

All diseases of the respiratory apparatus, which tend to diminish the aerating surface either organically or functionally, will produce a corresponding increase in the number of the respiratory movements, according to the extent of the disease. Exercise, fever, local inflammation, etc., will have this same effect.

In order to appreciate the respiratory effort we must get the simplest idea of the thorax.

The equine thorax is like a box, bounded superiorly by the eighteen dorsal vertebræ; laterally, by the eighteen ribs (on each side) and the interspaces filled by the intercostal muscles; inferiorly, by the sternum; posteriorly, by the diaphragm; and anteriorly by the important vessels, the œsophagus, the lymphatic glands and the trachea. Roughly speaking, this is a box which has but one entrance, and that is through the trachea.

As you look at the healthy thorax during inspiration you notice a slight movement of the ribs outward and forward.

The anterior ribs move least; in fact, the first rib does not move any; the second rib has a slight movement; the third moves more, and so on, the amplitude of their movements increasing up to the ninth, which is most movable. The motion then diminishes gradually to the eighteenth rib.

In your examination you notice first that there is movement of the ribs or chest wall, then whether these movements are full or shallow, and thirdly, whether one side moves more or less freely than its fellow.

In some cases you will be compelled to carefully observe whether the diaphragm does most of the work, or whether the ribs do their share or not.

We notice in a given case a decided diminution, or even a total loss, of motion of one side of the chest. What may produce this? What are the conditions which will cause such a pronounced symptom? First, Pleurodynia, a rheumatic affection of the intercostal muscles, may cause it; but the prominent cause is a spot of inflammation on the surface of that delicate serous membrane, the Pleura. This membrane, as you know, is a closed sac with its two surfaces, the visceral layer covering the lung and the parietal layer spread over the chest wall, lying in contact one with the other.

In health during an expansion of the chest cavity with a consequent distention of the lungs the two surfaces of this delicate layer play upon one another without causing any sensation whatever. As soon, however, as inflammation begins, no matter from what cause, whether intrinsic or extrinsic, we have immediately following the congestion of the part a desquamation, or falling off, of the endothelial cells, the protecting layer which covers the plexuses of sensitive nerves and distended blood vessels which constitute an important layer of the pleura. This is the first stage of Pleurisy. The exposure to friction, during the respiratory movements of these delicate nerve fibres which are already suffering from the pressure of distended capillaries, gives intense and instantaneous pain. This the animal avoids or diminishes by favoring that side, by holding the chest wall quiet and thus preventing the rubbing of this sensitive surface, which would otherwise be exceedingly painful. The animal will in many cases attract your attention by *pointing* repeatedly to the painful spot. Consequent upon diminished movement of this affected side the lung can do but little work, and its fellow must act vicariously and thus do double duty. As a result we have increased movement of the healthy side.

In certain forms of Subacute Pleurisy, such as the variety called "Pleurisy with Effusion," we have exuding from the vessels a quantity of serum which falls down to the lower portion of the pleural cavity, separating in this way the two layers, parietal and visceral, of the pleura.

If the amount of effused serum be considerable we will notice on inspection besides loss of motion, a decided bulging of that side. If the fluid be great enough to compress the lung, that is, occupy a space formerly filled by lung tissue which can now collapse, we may notice a decided swelling out of the affected side, with widening of the intercostal spaces and bulging of the same, with displacement of adjoining viscera.

In cases of Laryngeal, or Trachael disease with obstruction to the entrance of air to the lungs, you will notice with every inspiratory effort a sinking in of the antero-sternal space, a hollowing of the intercostal spaces and marked retraction of the abdominal wall.

The second method employed in Diagnosis is Palpation. This is simply the act of laying on the hand and feeling the external surface of the body. It has a wider range than Inspection. It brings us nearer our patient and is more useful in determining the amount of local expansion, and the character of vibrations or impulses communicated to the external surface.

In certain cases of Acute Pleurisy, where we have the delicate smooth surface changed to a roughened one, as I briefly described a few moments ago, the movements of the lung although slight, still allow during inspiration, a rubbing of this surface. When we place our hand over this spot we feel a peculiar sensation communicated to it during the inspiratory act, called the friction fremitus. It can only be appreciated in the first stage of pleurisy before the surface has been protected by the plastic exudation, nature's process of recuperation.

Again; if in a case of pleurisy you make firm pressure in the intercostal space over the seat of the inflammatory process, the animal will wince, grunt, or even groan.

Under this heading we must also consider those results of palpation obtained by feeling the pulsation of the arteries.

The pulse is best examined in the horse by feeling the submaxillary artery on the inner surface of the lower jaw, while in the dog and cat the crural artery on the inner side of the thigh will be easiest found.

The natural pulse of the horse after a period of rest beats about forty times a minute; that of the cow from forty to fifty; and the pulse of the dog ranges from eighty to one hundred, depending upon the size and breed of the animal. The pulse of the sheep ranges from seventy to eighty per minute.

What is the pulse? How is it produced?

By a contraction of the left ventricle of the heart a volume of blood is forced out into the first portion of the aorta. You all no doubt have learned the mechanism of the pulse from the lectures of your esteemed Professor of Physiology, and I only wish in a few words to bring the subject once more to your attention. As the aorta receives its new complement of blood it becomes distended; but on account of its elasticity, immediately following this distension there comes a contraction. This dilatation with its accompanying contraction is propagated in a wavewhich passes all along the whole arterial tree through its minutest subdivisions.

While the wave passes instantaneously along the vessels, the blood requires an appreciable space of time for its transmission.

The impulse communicated to our finger then is synchronous, that is, it occurs at the same time, with the contraction of the left ventricle.

This is sometimes of great assistance in our examinations of the heart. In certain diseased conditions it is difficult to determine which is the systolic or first sound. If we recollect the fact just stated we will place the finger on an artery and that cardiac sound which is synchronous with the pulse, must be the systolic sound.

The pulse has been the subject of much study, but it was not until the celebrated Marey invented the sphygmograph, that our knowledge of its physiological character was very definite. * * * * * * *

By those who would carefully study the pulse in its relation to disease, six important characteristics will be observed. These are; first, the force; second, the frequency; third, the rythm; fourth, the size; fifth, the quality; and sixth, the duration.

This division with the several characteristics may seem like an extra refinement to those of you who have never considered the pulse except in regard to its frequency; but we think there are none here, who can not, if they are observant and persevering, in a short time appreciate these apparently fine shades of distinction.

How may one pulse differ from another?

In all these points? Yes. That is one pulse may be strong, may beat forcibly upon the finger, and another may be weak and feel but feeble beneath our touch. One pulse may be rapid or frequent as it is called, and another may be slow or infrequent. One may be regular, another irregular or intermitting. By regular we mean the same number and kind of beats to the minute.

An intermitting pulse is one in which we skip a beat now and again; some times this loss of a beat is regular, that is, it occurs every five, ten, or other number of pulsations, or is irregular. One pulse may be large, another may seem quite small.

One may feel hard like a cord beneath the finger, another may be so soft we can compress it and with a little pressure prevent the pulsation in the vessel beyond.

Last of all we may notice that one pulse seems to delay and last longer beneath the finger, than another which appears to depart as quickly as it came.

So we have strong or weak pulses, rapid or slow pulses, those which are regular, irregular, or intermitting, large or small pulses, hard or soft pulses, and finally, long or short pulses.

These terms are as you see only comparative.

We consider the pulse which is noticed after exercise in health as our standard and it possesses the following qualities.

It is a strong, frequent, regular, large, medium hard, and long pulse.

A typical case of Pneumonia is accompanied by a strong, frequent, regular, large, soft, and short pulse.

Inflammations of serous membranes and notably of the peritoneum are associated usually by a pulse which is strong, frequent, regular, small, hard and short. This has been called a cordy, or wiry pulse.

Anaemia due to recent profuse hemorrhages has frequently a weak, frequent, regular, large, very soft, and short pulse. This has been called a gaseous pulse; because it gives you the sensation of a bubble of gas passing beneath the finger.

Acute Laminitis is associated with a strong, frequent, regular, large, hard, and long pulse.

In hypertrophy of the left ventricle of the heart we frequently find a pulse which is strong, infrequent, regular, large, hard, and long. * * * * * *

Observe how each of the conditions above mentioned has a pulse which possesses distinctive features, which are not matters of theory only; for you will be able to appreciate their importance as you see more and more of it verified by your experience.

Intermission of the pulse may occur as the result of indigestion; and without other symptoms it is not important. Animals with this kind of a pulse do not usually stand diseases well.*

*Williams.

